# Reliability of shear wave elastography for the assessment of gastrocnemius fascia elasticity in healthy individual

**DOI:** 10.1038/s41598-022-12786-1

**Published:** 2022-05-24

**Authors:** Jiping Zhou, Yuyi Lin, Jiehong Zhang, Xingxian Si’tu, Ji Wang, Weiyi Pan, Yulong Wang

**Affiliations:** 1 Shenzhen Dapeng New District Nan’ao People’s Hospital, Branch of the First Affiliate of Shenzhen University, No.6 Renmin Road, Nanao street, Dapeng New District, Shenzhen, Guangdong Province China; 2 Clinical Medical College of Acupuncture, Moxibustion and Rehabilitation, Guangzhou University of Chinese Medicine, Guangzhou, China; 3 The First Affiliated Hospital of Shenzhen University, Shenzhen Second People’s Hospital, No. 3002, Sungang West Road, Futian District, Shenzhen City, Guangdong Province China

**Keywords:** Rehabilitation, Medical imaging

## Abstract

The mechanical properties of the deep fascia, particularly their stiffness, strongly affect the development of muscle pathologies (such as compartment syndrome) and the action of the muscles. However, the mechanical characteristics of the deep muscular fascia are still not clearly understood. The present study focuses on examining the reliability of ultrasonic shear wave elastography (USWE) devices in quantifying the shear modulus of the gastrocnemius fascia in healthy individuals, particularly their ability to measure the shear modulus of the deep fascia of the gastrocnemius during ankle dorsiflexion. Twenty-one healthy males (age: 21.48 ± 1.17 years) participated in the study. Using USWE, the shear moduli of the medial gastrocnemius fascia (MGF) and lateral gastrocnemius fascia (LGF) were quantified at different angles during passive lengthening. The two operators took turns measuring each subject’s MGF and LGF over a 1-h period, and operator B took an additional measurement 2 h later. For the intra-operator test, the same subjects were measured again at the same time of day 5 days later. Both the intrarater [intraclass correlation coefficient (ICC) = 0.846–0.965)] and interrater (ICC = 0.877–0.961) reliability values for measuring the shear moduli of the MGF and LGF were rated as excellent; the standard error of the mean (SEM) was 3.49 kPa, and the minimal detectable change (MDC) was 9.68 kPa. Regardless of the ankle angle, the shear moduli of the LGF were significantly greater than that of the MGF (*p* < 0.001). Significant increases in the shear moduli of both the MGF and the LGF were observed in the neutral position compared to the relaxed position. These results indicate that USWE is a reliable technique to assess the shear modulus of the gastrocnemius fascia and detect its dynamic changes during ankle dorsiflexion. USWE can be used for biomechanical studies and intervention experiments concerning the deep fascia.

## Introduction

The deep fascia of the gastrocnemius (GDF), which serves as a structural support and an anatomical boundary, is the main structure that maintains the general shape of this muscle to build up support and prevent adhesion between muscles^1^. The primary function of the GDF is to provide and transmit forces to connective tissue, thereby regulating human posture and movement^2^. Previous studies have shown that stress is repeatedly transmitted to the deep muscular fascia during relaxation (e.g., dynamic stretching) and activation (e.g., jumping or running) of the extremity muscles. The deep fascia may be remodeled to accommodate this stress, potentially reducing the inherent stiffness of these tissues and preventing or reducing tissue damage^3^. Anatomical studies demonstrate that the GDF is composed of three layers of connective tissue with different orientations and densities^4,5^. This fascia connects to the underlying muscles^5^, which not only augment the ability of fascial tissues to bear strain (including the force produced by single- and multiple-muscle contraction) in all directions but also transmit the forces to adjacent tendons or muscles in an effective manner^6^. From the perspective of biomechanics, the deep fascia plays an important role in movement restriction and proprioception^3,7^. This restrictive role has been observed in fascia connection models and cadaveric models, such as connections between the deep fascia of the medial gastrocnemius and the pelvis^8,9^. This also means that when fascia tissue is stretched in one part, it may cause restriction, tension or pain in other parts of the body^10^. Previous studies have shown that the muscle fascia is an uninterrupted viscoelastic membranous tissue that can modify muscles’ elasticity. Lessening the stiffness of the fascial tissue in the transverse direction not only contributes to maintaining intramuscular pressure but also allow radial expansion of the underlying muscle^3,5,11^. The increase in GDF stiffness is associated with pain or soreness of the gastrocnemius (e.g., gastrocnemius myofascial pain), even muscle injuries^1^. The application of soft tissue manipulation can relax the fascia, thereby improving joint flexibility and decreasing pain^12^. However, we found no studies in which the elasticity of the GDF had been measured in vivo. Therefore, a detection technique to quantify the shear modulus of the GDF in a reliable, quick and objective manner may provide useful information for the development of adapted recovery strategies and curative effects of fascia therapy.

The shear modulus of the GDF can be assessed with techniques and methods such as palpation and magnetic resonance elastography (MRE)^13,14^. Palpation, used by clinicians to assess whole soft tissue stiffness, is a useful and valuable tool, but it merely provides qualitative information about tissue stiffness (soft versus hard) and is reported in a dichotomized manner (presence or absence of stiffness). MRE requires subjects to remain in place for a long time and restricts their position during measurement. Thus, these methods and techniques cannot meet the requirements of dynamic and quantitative monitoring of the shear modulus of GDF performance. Dynamic monitoring of the mechanical properties of the fascia contributes to improving diagnostics as well as monitoring disease progression and treatment response^15^. Ultrasonic shear wave elastography (USWE) is an effective quantitative technique to evaluate the elastic properties of soft tissues, such as muscle, tendon, and fascia^4,16–19^. USWE can be used to estimate the elastic properties of a local target area through shear wave speed; in this way, insight will be gained into how the body responds to various forces and treatments. The elastic properties of soft tissues have been measured using USWE in various conditions (e.g., at rest, before and after stretching, during and after exercise)^16–19^. USWE is more suitable than MRE for use in the contexts of sports medicine and rehabilitation.

Our previous studies have shown that USWE is an effective and reliable technique for estimating the elastic properties of muscles and tendons, such as the gastrocnemius and Achilles tendon, and reflecting the biomechanical properties of muscles and tendons ^18^. In addition, USWE was used to evaluate the passive behavior of plantar flexors during passive dorsiflexion^20^. They found that the shear modulus of the lower leg is inhomogeneous between muscles and displays differences during passive dorsiflexion^20^. However, USWE has not been used to quantify the shear modulus of the GDF, including the changes in its shear modulus during passive dorsiflexion. In addition, to accurately reflect ongoing disease progression or therapeutic effects in clinical testing, diagnosis and treatment, subjects must usually undergo multiple evaluations by two or more testers, such as different researchers, doctors or therapists. Therefore, it is important to estimate the reliability of USWE within and between raters). Unfortunately, we are not aware of any study examining the reliability of USWE for the measurement of GDF elasticity. To collect valid and reliable data in both clinical and research contexts, it is necessary to determine the degree of consistency and agreement regarding quantitative USWE measurements.

In summary, the primary objective of this study was to estimate the intra- and interrater reliability of USWE in quantifying the shear modulus of the GDF at different angles during passive lengthening. The operators took turns measuring each subject’s MGF and LGF over a 1-h period, and Operator B took the same measurement 2 h after the end of operator A's measurement. In the intra-operator test, the same subjects were measured again at the same time of day 5 days later. As a secondary objective, the change in the elastic properties of the GDF at various ankle angles was also investigated. We hypothesized that good consistency would be obtained when USWE was used to measure GDF elasticity, and the shear modulus of the GDF would increase with ankle dorsiflexion and that, regardless of the ankle angle, the shear modulus of the MGF would be significantly greater than that of the LGF.

## Methods

### Ethical approval

This study was approved by the Ethics Committee of Luoyang Orthopaedic Hospital, Henan Province (No. KY2019-001-01). The present study follows the principles of the Declaration of Helsinki. All participants were fully informed of the relevant features of the study, such as purpose and process, and signed a written informed consent form.

### Subjects

Twenty-one male volunteers with history of lower limb injury were invited to participate in this study. This study was performed at Luoyang Orthopaedic Hospital in Henan Province, China.

### Equipment

The procedures for muscle fascia shear modulus measurement were similar to those in our previous studies^16–19^. The equipment was an ultrasonic instrument (Aixplorer Supersonic Imagine, version 6.0, Aix-en-Provence, France) with built-in shear wave elasticity imaging technology, and a 40-mm linear-array transducer (SL15-4) was used to capture USWE images and quantify the shear moduli of MGF and LGF). The AixPlorer ultrasonic scanner was set to the following settings. Maps of the shear modulus were obtained at 12 Hz. The shear wave elastography mode was musculoskeletal mode. The USWE option was set to penetration mode. The opacity was 85%. The gain was 90%. The smoothing level was 5. The persistence setting was off. The shear modulus ranged from 0 to 800 kPa. The B-scan depth was 3.0 cm^16,17^. The Q-box diameters of the MGF and LGF were set to 1 mm. The size of each region of interest (ROI) was set to 10 × 10 mm^18^, and the ROIs were positioned along the longitudinal sections of the MGF and LGF^16,19^.

### Procedures

Only the dominant leg of each participant was studied^16,17,19,20^, and participants were asked to rest for 10 min before testing. In addition, participants were asked to lie down in a prone position on the treatment bed, with their feet fully extended and projecting slightly beyond the edge of the bed, their knees fully extended, and their upper limbs naturally placed along the sides of the body^16^. A customized, movable knee–ankle–foot orthosis was used to fix the ankle. The shear modulus of the MGF and LGF was quantified at neutral position (90°) and relaxed position of the ankle joint. The neutral position representing the ankle joint was fixed at the neutral anatomical position, while the relaxed position representing the ankle joint was fully relaxed^16,17,19,20^. The angle of the ankle joint was measured with a handheld goniometer. To ensure that the ankle joint angles of subsequent repeated measurements were consistent with the initial measurements, the exact angle of the ankle joint in the relaxed position was recorded after the first position. The shear moduli of the MGF and LGF were measured on the medial and lateral sides, respectively, in the proximal 30% of the region between the calcaneus and the popliteal fossa. Length was measured with a tape measure^16,17,19,20^. The placement direction of the scanner was parallel to the line connecting the calcaneus and the medial or lateral popliteal fossa. To ensure identical scanner placement in all USWE measurements, the measurement location and direction of the scanner were marked with waterproof markers. For the accuracy of the experimental measurements, participants refrained from high-intensity exercise for 48 h before testing, and they were asked to keep their bodies fully relaxed throughout testing.

**Figure 1:**
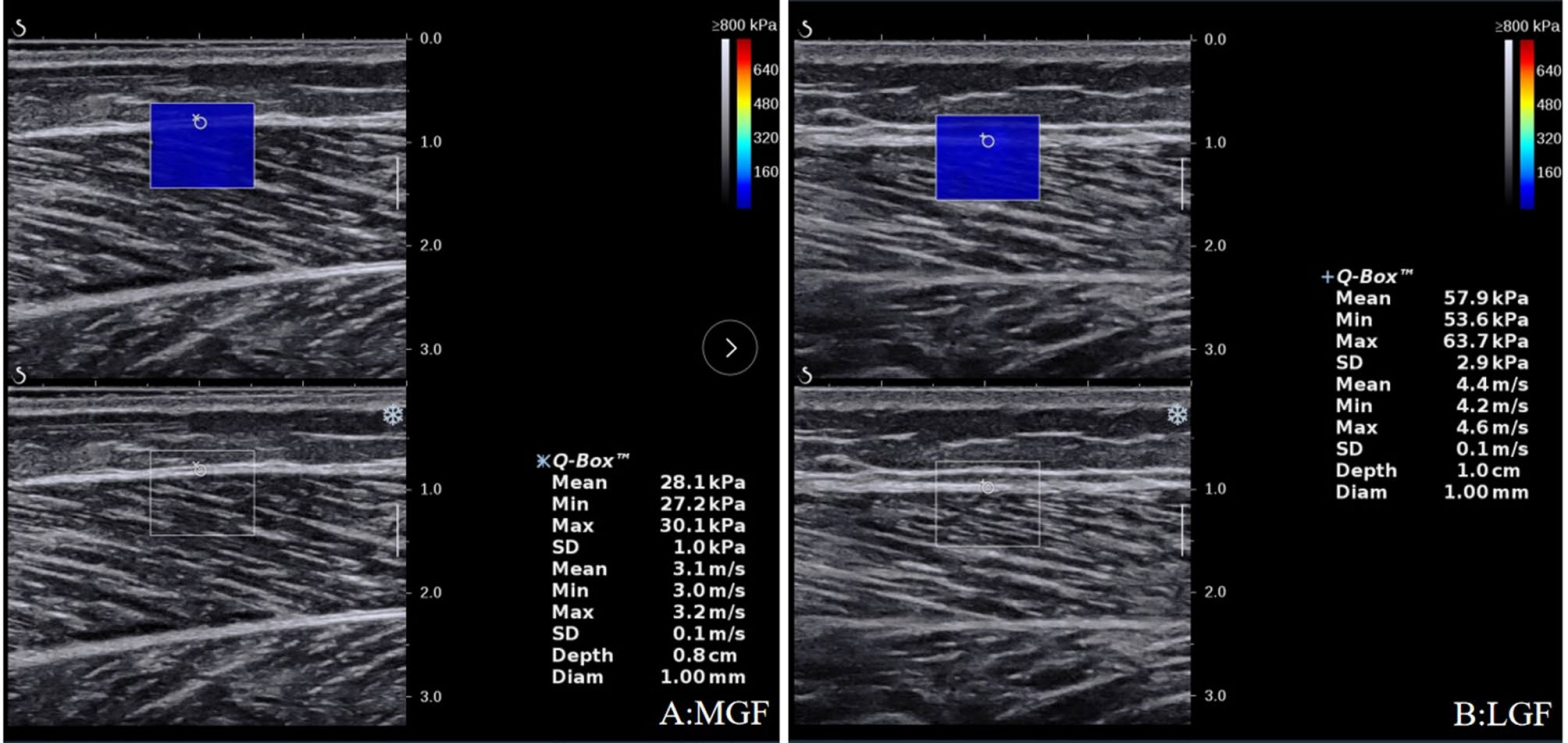
Typical maps of the elastic properties of the MGF and LGF in the longitudinal directions. Color-coded boxes representing muscle fascia elasticity are shown in the upper images. The longitudinal grayscale sonograms of muscle fascia are shown in the bottom images. The measurement of shear modulus is performed in a circle. The Q-Box™ is shown on the right. MGF: medial gastrocnemius fascia; LGF (**B**): lateral gastrocnemius fascia.

All participants received a USWE examination from experienced physical therapists (P.W.Y. and Z.J.P.) with 4 years of experience performing ultrasonography. In addition, the USWE examination was supervised by a sonographer (S.T.X.X) with 13 years of experience. The shear modulus was quantified with an AixPlorer ultrasonic scanner positioned on the skin markers in the neutral position and relaxed position of the ankle joint. To ensure that the muscle and tendon regained their original elastic properties and to relieve the tension on the GDF between angle switching, the shear modulus at each joint angle was measured at 5-min intervals^22^. First, as described in our previous studies^23–25^, we applied sufficient ultrasound gel to the skin markers. Second, the midpoint of the transducer was placed in the markers, and the B-mode was activated to ensure that the muscle belly was assessed and then rotated in a longitudinal orientation until the grayscale image displayed the appearance of the muscle (Fig. 1). Third, the mode of USWE was activated, the transducer was kept motionless for more than 8 s, and the image was frozen until the color in the ROI was uniform and several fibers were continuously visible^16,17,19,20^. Three images were captured at each measurement site of muscle fascia. Image quality was closely monitored throughout all measures.

Two operators (A and B) took part in the inter-operator investigation. The operators took turns measuring each subject’s MGF shear modulus and LGF shear modulus according to the aforementioned program and by operator B with a 2-h interval. In the second test, the same subjects participated at the same time 5 days later, which was repeated by operator A for the intra-operator test. Subjects were asked to maintain their normal activity but avoid high-intensity physical activities, such as long-distance running^26^. The measurement results of each subject were recorded by L.Y.Y.

### Data analysis

Statistical analysis was performed using SPSS Version 19.0 (SPSS, Chicago, IL). All data are expressed as the mean ± standard deviation. The normality of the data was tested by the Shapiro−Wilk test. The intra- and interrater reliability was evaluated by calculating the intraclass correlation coefficient (ICC). The intrarater (measurements taken on 2 occasions separated by 5 days) and interrater (measurements by 2 operators) reliability were examined using ICC (3,1) and ICC (3,2)^26^. The standard error of the mean (SEM) was calculated by the formula SEM = standard deviation × √(1 − ICC), the coefficient of variation (CV) was calculated by the formula CV = (standard deviation/mean) × 100%, and the minimal detectable change (MDC) was computed by the formula MDC = 1.96 × SEM × √2. ICC values < 0.50 are classified as poor, values between 0.5 and 0.75 are moderate, values between 0.75 and 0.9 are good, and values greater than 0.9 are excellent^27^. Pearson’s correlation was used to examine the relationship between the shear modulus muscle fascia and the patients’ demographic information (age, height, weight, body mass index (BMI), and weekly exercise hours). Muscle fascia shear modulus values were analyzed using general linear model nested ANOVA. The fixed effects followed a two-way factorial treatment structure, ankle angle × muscle fascia. The random effect model was the ankle angle nested within the muscle fascia. Based on the overall p value from the ANOVA (*p* < 0.05), a post hoc assessment using Tukey’s test was subsequently performed to make pairwise comparisons. In addition, the effect size was calculated using Cohen’s d. *p* < 0.05 was considered significant.

## Results

### Demographic data

Thirty male individuals were screened for study participation. Nine were excluded because they did not meet the study criteria. Demographic and anthropometric variables (including age, height, weight, BMI, and weekly exercise hours) for the remaining 21 enrolled participants are summarized in Table 1. Age, height, weight, BMI, and weekly exercise hours were not significantly associated with shear modulus in either the MGF or the LGF (all *p* > 0.05).

**Table 1:** Demographic information of participants (N = 21 male subjects).

Characteristic	Mean ± SD
Age (years)	21.48 ± 1.17
Weight (kg)	61.25 ± 5.57
Height (m)	1.71 ± 0.05
BMI (kg/m^2^)	20.85 ± 1.84
Weekly exercise hours	4.30 ± 1.56

SD, standard deviation. BMI, body mass index.

### Intra- and interrater reliability of the shear moduli of the MGF and LGF

**Figure 2:**
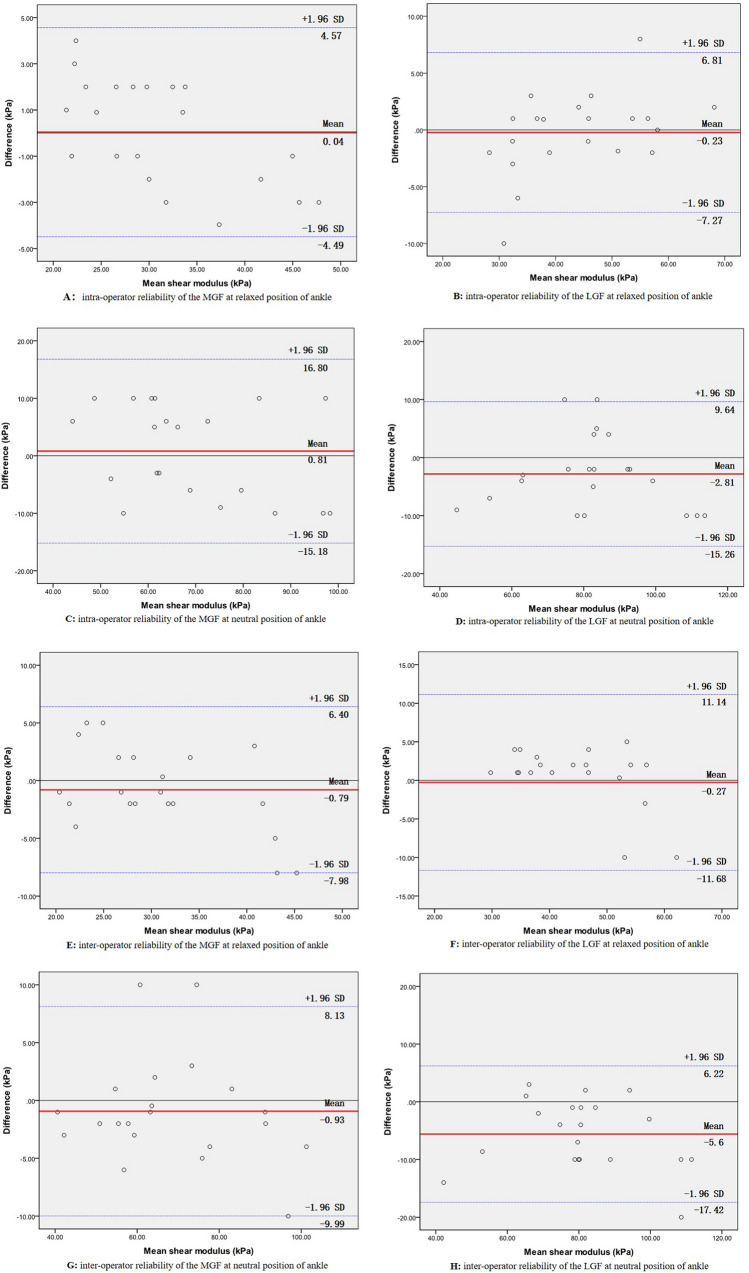
Bland–Altman plots of intra- and inter-operator reliability of the shear moduli of the MGF and LGF. The differences in the shear moduli between day 1 and day 5 are plotted against the mean of each participant for the MGF (**A**: relaxed position; **C**: neutral position) and LGF (**B**: relaxed position; **D**: neutral position). The differences in the shear moduli between operator (**A**) and operator (**B**) are plotted against the mean shear moduli of each participant for the MGF (**E**: relaxed position; **G**: neutral position) and LGF (**F**: relaxed position; **H**: neutral position). In each image, the continuous line is the mean difference, and the dotted lines represent two SDs above and below the mean difference.

The ICC, MDC, CV, and SEM for intra- and interrater reliability for the mean shear moduli of the MGF and LGF can be found in Table 2. In the relaxed ankle position, the mean shear moduli of the MGF and LGF, respectively, were 31.16 kPa and 43.93 kPa for operator A in test 1 and 31.20 kPa and 43.70 kPa for operator A in test 2, and 30.37 kPa and 43.66 kPa for operator B. In the neutral ankle position, the mean shear moduli of the MGF and LGF, respectively, were 68.79 kPa and 84.02 kPa for operator A in test 1, 69.60 kPa and 81.21 kPa for operator A in test 2, and 67.86 kPa and 78.42 kPa for operator B. The intrarater (ICC = 0.846–0.965) and interrater (ICC = 0.877–0.961) reliability were good to excellent for the shear moduli of the MGF and LGF^15,17,28^. The SEM (kPa) was 1.41 to 3.49, the MDC (kPa) was 3.92 to 9.68, and CV (%) was 21.50 to 27.69. Bland–Altman plots of the intra- and inter-operator reliability values of the relaxed ankle position are shown in Fig. 2A,B,E and F. The mean differences were 0.04, − 0.23, − 0.79 and − 0.27 kPa, respectively, and the 95% limits of agreement were − 4.49–4.57 kPa, − 7.27–6.81 kPa, − 7.98–6.40 kPa and − 11.68–11.14 kPa, respectively. Other plots of intra- and inter-operator reliability in the neutral ankle position are shown in Fig. 2C,D,G and H. The mean differences were 0.81, − 2.81, − 0.93 and − 5.6 kPa, respectively, and the 95% limits of agreement were − 15.18–16.80 kPa, − 15.26–9.64 kPa, − 9.99–8.13 kPa and − 17.42–6.22 kPa, respectively.

**Table 2:** Intra- and inter-tester reliabilities of USWE for mean shear modulus of MGF and LGF.

	Measurement position	Ankle angle	Test 1 (kPa)	Test 2(kPa)	MDC (kPa)	CV (%)	SEM (kPa)	ICC (95%CI)
Intra-tester	MGF	R	31.16 ± 8.95	31.20 ± 7.51	4.16	24.07	1.50	0. 896 (0.762–0.957)
		N	68.79 ± 17.77	69.60 ± 15.07	8.35	21.65	3.01	0.965 (0.915–0.985)
	LGF	R	43.93 ± 10.51	43.70 ± 12.10	6.71	27.69	2.42	0.846 (0.659–0.935)
		N	84.02 ± 18.15	81.21 ± 17.46	9.68	21.50	3.49	0.941 (0.862–0.976)
Inter-tester	MGF	R	31.16 ± 8.95	30.37 ± 7.07	3.92	23.28	1.41	0.961 (0.907–0.984)
		N	68.79 ± 17.77	67.86 ± 16.98	9.41	25.02	3.40	0.877 (0.723–0.948)
	LGF	R	43.93 ± 10.51	43.66 ± 10.46	5.80	23.96	2.09	0.950 (0.881–0.979)
		N	84.02 ± 18.15	78.42 ± 17.05	9.45	21.74	3.41	0.936 (0.850–0.974)

*R* Relaxing position, *N* Neutral position.

*LGF* Lateral gastrocnemius fascia, *MGF* Medial gastrocnemius fascia.

*MDC* Minimal detectable change, *CV* Coefficient of variation, *SEM* Standard error in measurement, *ICC* Intra-class correlation coefficient.

### Changes in the shear moduli of the MGF and LGF

**Figure 3:**
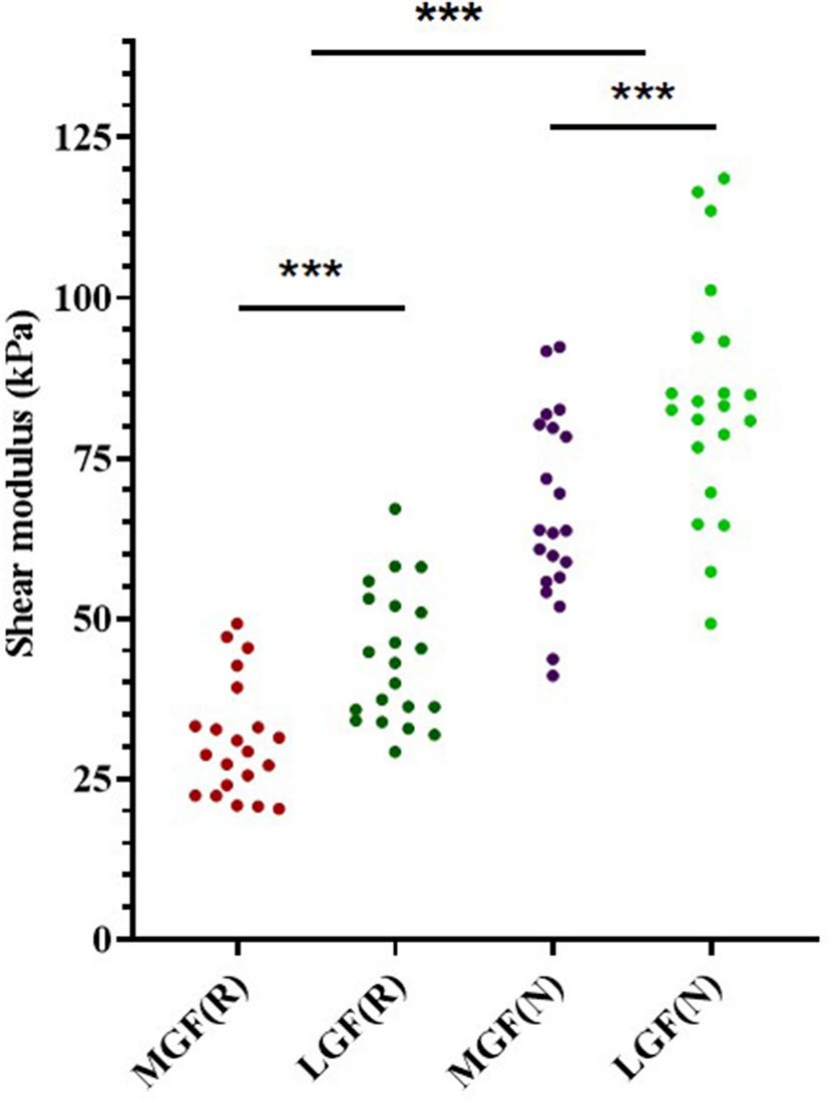
Variations in the elasticity of the MGF and LGF of the passive ankle joint in the relaxed position and the neutral position with the knee fully extended. *** *p* < 0.001.

Regardless of the ankle angle, the shear modulus of the LGF (relaxed position: 40.98%; neutral position: 22.14%) was significantly greater than that of the MGF (effect size: 0.85; 95% CI: 70.38–82.43; *p* < 0.001; Fig. 3). Significant increases in the shear moduli of both the MGF (120.76%) and the LGF (91.26%) were observed in the neutral position compared to the relaxed position (effect size: 2.70; 95% CI: 56.18–71.77; *p* < 0.001, Fig. 3).

## Discussion

The present study is the first to document that USWE is a reliable technique to quantify changes in the shear modulus of the GDF. The good (0.90 > ICC > 0.75) to excellent (ICC > 0.90) intra- and interrater reliability of measurements made using USWE and the relatively low values of MDC and SEM support the accuracy of the assessment. The shear modulus of the GDF (including the LGF and MGF) increased significantly when the ankle was in a neutral position compared to the relaxed position. Furthermore, regardless of the ankle angle, the shear modulus of the LGF was significantly greater than that of the MGF.

This is the first study to document excellent intra- and interrater reliability for the elastic properties of the GDF (including LGF and MGF) using USWE in healthy males. Previous studies of the same type merely investigated the reliability of the elastic properties of muscles, fascia and tendons using the USWE^4,21,29^. For example, Otsuka et al. (2019) used USWE to investigate in the mechanical properties of the deep fascia due to muscle contraction; they found high reliability for the fascia lata in the longitudinal direction (ICC = 0.618–0.989)^4^. Le et al. used USWE to quantify the elastic properties of the gastrocnemius muscle during passive dorsiflexion with excellent reliability (ICC = 0.92–0.96)^21^. Saeki et al. (2017) studied the reliability of USWE in assessing the elastic properties of the medial and lateral gastrocnemius at three different dorsiflexion angles^29^. Their findings revealed that the reliability of USWE at various ankle angles was excellent (ICC = 0.76–0.91). This means that the USWE was able to reliably measure the changes in both the medial and lateral parts of this muscle. However, the elastic properties of the MGF and LGF were not measured. The findings of the present study were similar to those of studies that have quantified the elastic properties of muscles using USWE. In addition to using ICC values to evaluate intra- and interrater reliability, our study also computed the SEM (1.41 to 3.49 kPa) to further verify reliability. The relatively low SEM values confirmed the precision of the MGF and LGF measurements.

We found that the interrater reliability (ICC = 0.846–0.965) appeared to be consistent with the intrarater reliability (ICC = 0.877–0.961) at the same site. These results are similar to previous reports. Chen et al. (2020) reported similar results in which the intrarater reliability (ICC = 0.860–0.938) was consistent with interrater reliability (ICC = 0.904–0.944) for measuring the elastic properties of the thoracolumbar fascia using the USWE^30^. This means that the measurement of gastrocnemius fascia using ultrasonic shear wave elastography is reliable and does not change with time or among different operators. In addition, our findings revealed that the CVs of the MGF and LGF ranged from 21.50% to 27.69%, which were slightly higher than those reported in previous similar studies. Lima et al. (2017) reported that the CV values of MG were 17.29% to 20.95% during rest, and a similar finding was observed by Chino and Takahashi (19.4%)^31^. This could be related to probe load or operator dependency, as the probe may not be completely parallel to the muscle fascia^23,32^. Another possible reason is the shear modulus range of the ultrasonic scanner. In this study, we set it to the maximum range (800kpa). This may decrease the resolution and thus also the differences across measurements/partecipants. However, as the present study found high reliability for the shear moduli of the MGF and LGF in the longitudinal direction (interrater ICC: 0.846–0.965; intrarater ICC: 0.860–0.938), the data for the deep muscular fascia were all classified as acceptable. Furthermore, we reported the MDC. From the clinical and experimental perspectives, the MDC, as the smallest statistically significant change in measurement results, can reflect the precision with which real change is detected and serve as a reference for future study. In terms of our results, the shear modulus of the GDF should be greater than 9.68 kPa to reflect real changes between repeated tests.

This study uncovered new findings regarding the shear modulus between MGFs and LGFs. Our findings showed significant increases in the shear moduli of both the MGF and the LGF in the neutral position compared to the relaxed position. In addition, the shear modulus of the LGF was significantly greater than that of the MGF. The difference in the shear modulus from the neutral position compared to the relaxed position was greater than the MDC (9.68 kPa), which revealed that the change in elastic properties from the relaxed position was caused by real change rather than experimental errors. The above results indicated that the degrees of stretching level of the MGF and LGF were not identical during passive ankle dorsiflexion. Differences in stretch between the MGF and LGF might be due to the differences in recruitment and size (such as cross-sectional area, volume, and rotation angle) of the MG and LG and may also be associated with their passive force–length relationships^24,33–35^. The actual examination of the functional significance and biomechanical effect of the apparent difference in elastic properties between the MGF and LGF is a very interesting research topic and may suggest modifications of traditional exercise protocols. However, it is beyond the scope of this study, as it would require a different research design. In addition, to our knowledge, no studies have reported the elastic properties of the GDF in healthy individuals; thus, it was difficult to compare our findings directly with the results of previous studies. However, previous similar results revealed that the passive tensile response of the elastic properties of the gastrocnemius muscle was similar to our results in the same location. Liu et al. (2021) found that the stiffness of the gastrocnemius medius increased as ankle dorsiflexion increased (ankle movement from 40° of plantar flexion to 30° of dorsiflexion)^15^. Le Sant et al. (2017) demonstrated an increase in the shear modulus of the lower leg muscles (including gastrocnemius muscle) during passive dorsiflexion performed with the knee fully extended^21^. These results are in line with the physiologic stiffening of the muscles to resist the force applied during passive stretching. Previous studies have shown that the fascia lata can act as a spring, contributing to myofascial force transmission, elastic energy storage, and limb stability^11,36^. Otsuka et al. found that the shear modulus of the fascia lata increased with passive mechanical stress, and the relative changes in the shear moduli were not identical between the fascia lata and the muscles^4^. Therefore, changes in the shear moduli of the muscle and its fascia could be associated with the risk of muscle/tendon injury^37^. Previous studies have suggested the existence of force transmission among fascia, muscle and tendons^38,39^. The USWE measurements performed in this study were focused on one targeted muscle fascia and provide an indirect assessment of its passive tension. The specific influence of the fasciae of synergist muscles should be estimated in future studies.

## Limitations

The present study has some potential limitations. First, this study was meant primarily to establish a method for assessing the shear modulus in the MGF and LGF. We only recruited male healthy subjects as a preliminary experiment. Further studies need to be conducted to evaluate the regional difference in the deep fascia elasticity in female participants or patients with muscle strain. Second, we did not use electromyography (EMG) to monitor MG and LG activity to ensure whether the muscle contracted during the experiment. However, every participant was asked to remain relaxed, and there was no sign of muscle contraction on real-time ultrasound images. Thus, we believe that each subject followed the oral instructions and remained relaxed. Third, the Q-box diameter were set as 1 mm. In further studies, we will improve our measurement method by using a long rectangular Q-box or 3–5 Q-boxes with a diameter of 1 mm, which can cover more deep fascia of gastrocnemius muscle as much as possible.

## Conclusions

USWE is a viable technique to estimate the shear modulus of the gastrocnemius fascia in young healthy male individual. A change in the shear modulus by more than 9.68 kPa can be considered a true change rather than an error. In addition, the shear modulus of the LGF was significantly greater than that of the MGF. Moreover, this technique is capable of detecting the change in the gastrocnemius fascia between the neutral and relaxed positions, which provides the possibility for further studies of the dynamic changes in this fascia.

## Data Availability

The datasets used and/or analysed during the current study available from the corresponding author on reasonable request.
